# PEG-Modified *tert*-Octylcalix[8]arenes as Drug Delivery Nanocarriers of Silibinin

**DOI:** 10.3390/pharmaceutics13122025

**Published:** 2021-11-27

**Authors:** Desislava Budurova, Denitsa Momekova, Georgi Momekov, Pavletta Shestakova, Hristo Penchev, Stanislav Rangelov

**Affiliations:** 1 Institute of Polymers, Bulgarian Academy of Sciences, 103 Acad. Georgi Bonchev St., 1113 Sofia, Bulgaria; h_penchev@polymer.bas.bg; 2 Department of Pharmaceutical Technology and Biopharmaceutics, Faculty of Pharmacy, Medical University—Sofia, 2 Dunav St., 1000 Sofia, Bulgaria; dmomekova@yahoo.com; 3 Department of Pharmacology, Pharmacotherapy and Toxicology, Faculty of Pharmacy, Medical University—Sofia, 2 Dunav St., 1000 Sofia, Bulgaria; gmomekov@gmail.com; 4 Institute of Organic Chemistry with Centre of Phytochemistry, Bulgarian Academy of Sciences, Acad. Georgi Bonchev St. Bldg 9, 1113 Sofia, Bulgaria; Pavletta.Shestakova@orgchm.bas.bg

**Keywords:** calix[8]arenes, silibinin, inclusion complexes, PEGylation, cytotoxicity

## Abstract

The hepatoprotective properties of silibinin, as well its therapeutic potential as an anticancer and chemo-preventive agent, have failed to progress towards clinical development and commercialization due to this material’s unfavorable pharmacokinetics and physicochemical properties, low aqueous solubility, and chemical instability. The present contribution is focused on the feasibility of using PEGylated calixarene, in particular polyoxyethylene-derivatized *tert*-octylcalix[8]arene, to prepare various platforms for the delivery of silibinin, such as inclusion complexes and supramolecular aggregates thereof. The inclusion complex is characterized by various instrumental methods. At concentrations exceeding the critical micellization concentration of PEGylated calixarene, the tremendous solubility increment of silibinin is attributed to the additional solubilization and hydrophobic non-covalent interactions of the drug with supramolecular aggregates. PEG-modified *tert*-octylcalix[8]arenes, used as drug delivery carriers for silibinin, were additionally investigated for cytotoxicity against human tumor cell lines.

## 1. Introduction

**Figure 1:**
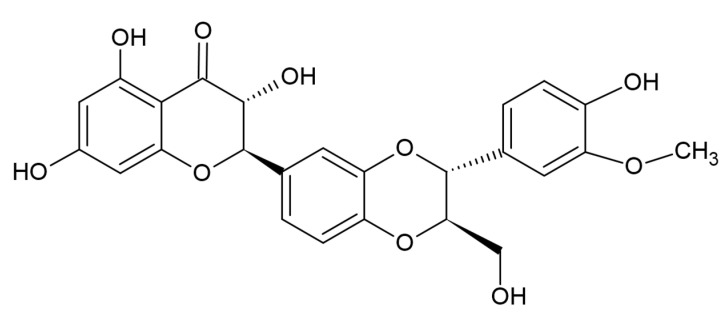
Chemical structure of silibinin.

The plant milk thistle (*Silybum marianum*) has been used since ancient times as a key element for various medical treatments. It has been effectively applied for curing gallbladder disorders and liver dysfunctions. Researchers’ findings have repeatedly claimed its effective hepatoprotective action [1]. The World Health Organization has in fact verified silymarin (a milk thistle derivative) as an established medicine [2]. As such, silymarin is present in silimonin, silychristin, silibinin, isosilychristin, isosilybin, and silydianin [3,4,5]. Silibinin (SBN), the main bioactive component, has proven its antioxidant properties and anticancer activity. It has been established that it possesses therapeutic effects by treating various malignancies, such as skin cancer [6], prostate cancer [7,8], breast cancer cells [9] and gastric tumor cells [10]. SBN is characterized by low bioavailability due to its high hydrophobicity and nonionizable chemical structure [11,12]. It is insoluble in apolar solvents and poorly soluble in water and polar solvents [13]. Its large structure, presented in Figure 1, further reduces its bioavailability and diffusion. Influencing molecules, such as phenol derivatives, amino acids and flavonoids, can improve SBN’s bioavailability [14]. To overcome these limitations and to mitigate the unfavorable pharmacokinetic profile, different nanoparticle-based drug delivery approaches are being developed to improve SBN bioavailability [15,16]. Systems based on polymeric nanoparticles demonstrate long-term stability, improved effectiveness, non-toxicity, and targeted drug release in comparison with traditional carriers [17,18,19,20]. In addition, when the particle size is under 200 nm, an increased drug accumulation in tumor cells is observed due to enhanced permeability [21]. 

Recently, extensive research efforts have been focused on supramolecules, such as crown ethers, cyclodextrins, and calix[n]arenes, due to their ability to encapsulate hydrophobic drugs through host–guest interactions [22,23,24,25]. The use of such macrocyclic platforms for the solubilization of purely water-insoluble, physiologically active substances is a synthetic approach to forming various types of amphiphilic molecules in a biomimetic way.

Since their discovery, a wide range of applications of calixarenes has been found due to their ability to entrap small molecules. This valuable feature has opened up many opportunities for the design and development of drug delivery. Calix[n]arenes, formed from phenolic units linked by methylene bridges at the 2,6-positions, can self-assemble into different ordered molecular aggregates. These supramolecular compounds have defined lower and upper rims and a large central cavity. They can form guest–host inclusion complexes through the encapsulation of small molecules and ions [26,27]. Calix[n]arenes’ most significant disadvantage is their low aqueous solubility. This issue has already been addressed via functionalization with polar substituents, such as sulfonates [28,29], phosphonates [30], amines, amino acids, peptides and saccharides [26,31,32,33], or poly(ethylene glycol) (PEG) [34,35]. 

In the present contribution, we are focused on the design of original PEG-modified *tert*-octylcalix[8]arenes and their evaluation as carriers for silibinin. In contrast to the more commonly used calix[4]arenes- and calix[6]arenes-based carriers, the products synthesized by us are characterized by functionalization with long substituents of both the lower and upper rims. The structure of calix[8]arene is identified by its considerably bigger cavity, which allows a higher load with larger molecules and aggregates. The attachment of tert-octyl groups in the upper rim forms a “crown” above the calixarene’s cavity through its side methyl-branched groups. This architecture additionally enlarges the actual molecule, and it is highly likely to enlarge the cavity volume of the calixarene basket, which in turn could lead to the inclusion of bigger molecules. The modification of the lower rim through PEG chains leads to the construction of a unique architecture of amphiphilic macromolecules, consisting of a hydrophobic *tert*-octylcalix[8]arene core and eight long arms of hydrophilic PEG chains. Although silibinin has been proven to be a very promising drug candidate, and it is classified as belonging to class II of the Biopharmaceutics Classification System (BCS) (drugs with high permeability and poor solubility), its bioavailability is limited by its poor dissolution and solubility. In this regard, the original PEG-modified *tert*-octylcalix[8]arenes were investigated in detail as a tool for improving the unfavorable aqueous solubility of silibinin. Additionally, both non-loaded and SBN-loaded complexes were investigated for cytotoxicity and against human tumour cell lines.

## 2. Materials and Methods

### 2.1. Materials

The PEGylated *tert*-octylcalix[8]arenes were synthesized as described in Section 2.2. Ethylene oxide was supplied by (Clariant, Muttenz, Switzerland). Silibinin, 1,6-diphenyl-1,3,5-hexatriene (DPH), xylene, potassium hydroxide, RPMI-1640 medium, L-glutamine and fetal calf serum (FCS) were purchased from Sigma-Aldrich (St. Louis, MO, USA). The cell lines HL-60 (chronic myeloid leukemia) and CAL-29 (transitional cell urinary bladder cancer) were purchased from the Leibniz Institute-DSMZ German Collection of Microorganisms and Cell Cultures (Braunschweig, Germany).

### 2.2. Synthesis of Amphiphilic PEGylated tert-Octylcalix[8]arenes

The synthesis of a series of products with different degrees of polymerization of the PEG chains is based on the process of the anionic polymerization of ethylene oxide (EO). *Tert*-octylcalix[8]arene was used as an initiator. The synthetic route, modified to suit the study purposes, was first described by Mustafina et al. [36]. Briefly, a mixture of p-*tert*-octylcalix[8]arene, KOH and xylene was placed into a three-necked flask and was heated to 140 °C under stirring in order to initiate azeotropic water evaporation. After water evacuation, the mixture was cooled to 110 °C. The synthetic route continued with the bubbling of ethylene oxide under a nitrogen atmosphere. The process was maintained for a set period of time in order to achieve PEG chains with the desired total degree of polymerization. The pH of the mixture was adjusted to pH 7 with 5% HCl. After filtration the solvent was evaporated. The product was taken up in dichloromethane and washed several times using deionized water. The solvent was removed under vacuum.

#### 2.2.1. ^1^H NMR and DOSY Characterization

The NMR spectra were acquired on a Bruker Avance II+ 600 NMR spectrometer equipped with a 5 mm direct detection dual broadband probe, and a gradient coil with maximum gradient strength of 53 G/cm. All spectra were measured at a temperature of 293 K. The DOSY (diffusion-ordered NMR spectroscopy) spectra were acquired with a convection-compensating double-stimulated echo-based pulse sequence, using monopolar gradient pulses (square shaped). The following experimental parameters were used: 32K time domain data points in the direct dimension (t2); 48 gradient strength increments; linear gradient ramp from 4 to 95% of the maximum gradient output (from 1.92 to 45.7 G/cm); 128 scans for each gradient step; relaxation delay of 2 s. To achieve optimal signal attenuation, experiments with different combinations of gradient pulse length, δ, (from 2 to 10 ms) and diffusion delay, Δ, (from 100 to 500 ms) were performed. The following parameters were used for DOSY spectra processing: 64 K data points in F2; exponential window function (line broadening factor 5); 258 data points in the diffusion dimension. The diffusion coefficients were calculated by fitting the diffusion profiles (the normalized intensity of selected signals as a function of the gradient strength G) with an exponential function using a variant of the Stejskal–Tanner equation adapted to the particular pulse sequence used. Assuming a spherical shape, the apparent hydrodynamic diameter, d_h_, of the particles was estimated using the Stokes–Einstein equation (Equation (1)) and the obtained value of the diffusion coefficient, *D*:

(1) $$d_{h}=\frac{kT}{3\pi \eta D}\;$$

where *k* is the Boltzmann constant, *T* is the temperature (K) and *η* is the solvent viscosity. In the present experiment, *η*(*D*_2_*O*) = 1.2518 × 10^−3^ Pa s at 293 K (NIST, Gaithersburg, MA, USA).

#### 2.2.2. Determination of the Critical Micellization Concentration (CMC)

A series of aqueous solutions of selected PEGylated *tert*-octylcalix[8]arenes with increasing concentrations from 0.008 to 4 wt. % were prepared. In total, 20 µL of a 0.4 mM solution of 1,6-diphenyl-1,3,5-hexatriene (DPH) in methanol was added to 2.0 mL of each of the polymer solutions. Afterwards, the solutions were vortexed briefly and left in the dark overnight. The spectra were recorded at 25 °C on a Beckman Coulter DU^®^ 800 spectrophotometer(Brea, CA, USA) in the wavelength interval 300–500 nm. The main absorption peak, characteristic for DPH solubilized in a hydrophobic environment, was at 356 nm.

### 2.3. Preparation of Inclusion Complexes of Silibinin and PEGylated tert-Octylcalix[8]arenes

#### Solvent Evaporation Method

For the preparation of inclusion complexes of silibinin and PEGylated *tert*-octylcalix[8]arenes and nanosized aggregates prepared thereof, a solvent evaporation method was chosen as previously described [23]. In brief, a series of samples containing a fixed concentration of SBN (1 mg/mL) and increasing concentrations of PEGylated *tert*-octylcalix[8]arenes (2–12 mg/mL) were prepared in absolute ethanol and evaporated to dryness using a Buchi rotation-type vacuum evaporator. The concentration range was chosen on the basis of the CMCs of the polymers to enable evaluation of their solubilizing capacity, both as a molecular solution (as inclusion complexes) and as a dispersion of supramolecular aggregates. Thereafter, the dried SBN:PEGylated *tert*-octylcalix[8]arenes-containing films were hydrated for 2 h with deionized water at 50 °C and then stirred for a further 24 h at ambient temperature in the absence of light. Afterwards, the undissolved silibinin was separated from the samples by centrifugation for 10 min at 5000 rpm. The clear colorless supernatants containing aggregates of SBN:PEGylated *tert*-octylcalix[8]arene complexes were quantified for SBN by UV–Vis spectroscopy at 286 nm. Phase-solubility graphs were obtained by the correlation of the amount of dissolved silibinin vs. the concentration of calixarenes.

### 2.4. Characterization of SBN:PEGylated tert-Octylcalix[8]arenes Inclusion Complexes and Supramolecular Aggregates

#### 2.4.1. Fourier Transform Infrared (FT-IR) Spectroscopy

The Fourier-transform infrared spectra (FTIR) of pure silibinin, pure PEGylated *tert*-octylcalix[8]arenes, their physical mixtures, and lyophilized inclusion complexes were measured in the range of 400–4000 cm^−1^ on an IRAffinity-1 FTIR spectrophotometer with a MIRacle Attenuated Total Reflectance Attachment (Shimadzu, Kyoto, Japan). The samples were analyzed in attenuated total internal reflection absorbance mode, with an aperture diameter of 3 mm and a spectral resolution of 1 cm^−1^. For an optimal signal-to-noise ratio, 50 scans were averaged per sample spectrum. All the spectra were normalized thereafter.

#### 2.4.2. Dynamic Light Scattering (DLS)

The size and size distribution patterns of silibinin-loaded supramolecular PEGylated *tert*-octylcalix[8]arenes aggregates were evaluated using a ZetaSizer NanoZS (Malvern Instruments, Malvern, United Kingdom), equipped with a 633 nm laser. The above-mentioned parameters were evaluated at the scattering angle of 175° at 25 °C. The hydrodynamic diameters (d_h_) were calculated using the Stokes–Einstein equation (Equation (1)) with η(H_2_O) = 0.890 × 10^−3^ Pa s at 293 K.

#### 2.4.3. Electrophoretic Light Scattering

The zeta potentials of silibinin-loaded supramolecular PEGylated *tert*-octylcalix[8]arenes aggregates were determined using a ZetaSizer NanoZS (Malvern Instruments, Malvern, United Kingdom), equipped with a 633 nm laser. The zeta potentials were evaluated at the scattering angle of 175° and 25 °C from the electrophoretic mobility using the Smoluchowski equation (Equation (2))

*ζ* = 4*πην*/*ε*, (2)

where *η* is the solvent viscosity, *ν* is the electrophoretic mobility, and ε is the dielectric constant of the solvent.

### 2.5. In Vitro Release Study

The cumulative release of silibinin from supramolecular OEC-IV and OEC-V aggregates was studied by membrane dialysis under physiologically relevant conditions, namely, 37 °C in acceptor media phosphate-buffered saline (PBS) at pH 7.4, since the possible route of administration of the tested formulations is parenteral. Briefly, 1 mL of each of the tested formulations was placed in a cellophane dialysis membrane tube (MWCO 10,000). The dialysis sacks were then placed in a temperature-controlled vessel in 100 mL PBS. The amount of acceptor phase was selected based on the solubility of silibinin, and thus, the chosen amount of dissolution media was able to dissolve more than 10 times the amount of SBN in the tested formulation. At predetermined time intervals, 2 mL aliquots were taken from the released medium and silibinin content was evaluated by UV–vis spectroscopy at λ = 286 nm from a liner curve (R^2^ = 0.9992) (liner eq. A = a + bx).

### 2.6. Cytotoxicity Evaluation

#### 2.6.1. Cell lines and Cultured Conditions

Human promyelocytic (HL-60) and urinary bladder cancer (Cal-29) cells were cultivated in RPMI-1640 culture medium, with the addition of 2 mM L-glutamine and 10% fetal calf serum, and were kept in an incubator (BB 16-FunctionLine’ Heraeus (Kendro, Hanau, Germany)) at 37 °C in a 5% CO_2_ humidified atmosphere.

#### 2.6.2. MTT Dye Reduction Assay

The cell growth inhibition potentials of free silibinin and its formulations were assessed using the MTT dye reduction assay. The method is based on the biotransformation of the yellow tetrazolium dye (MTT) to a violet formazan product via the mitochondrial succinate dehydrogenase in viable cells. The procedure was performed as described elsewhere [37] with small modifications [38]. Exponentially growing cells were plated in 96-well flat-bottomed microplates (100 μL/well) at a density of 3 × 10^5^ cells/mL (HL-60) or 1.5 × 10^5^ cells/mL (Cal-29), and after 24 h incubation at 37 °C they were treated with increasing concentrations of a silibinin-free drug (as ethanol solution) or loaded into supramolecular aggregates of PEGylated *tert*-octylcalix[8]arenes for 72 h. For each of the tested formulations a series of 8 wells was used. After the treatment time, samples of 10 µL of MTT solution (10 mg/mL in PBS) were added to each well. Afterwards, the microplates were incubated for an additional 4 h at the same temperature. Then, a 100 µL solution of 5% formic acid in 2-propanol was added to each well to dissolve the formed MTT–formazan crystals. The MTT–formazan absorption was evaluated at 580 nm with a Beckman-Coulter DTX800 multimode microplate reader (Brea, CA, USA). Thereafter, the fractions of surviving cells were calculated as a percentage of the untreated control. The half-inhibitory concentrations (IC50) were calculated from the concentration–response curves.

## 3. Results and Discussion

### 3.1. Synthesis of Amphiphilic tert-Octylcalix[8]arenes

**Figure 2:**
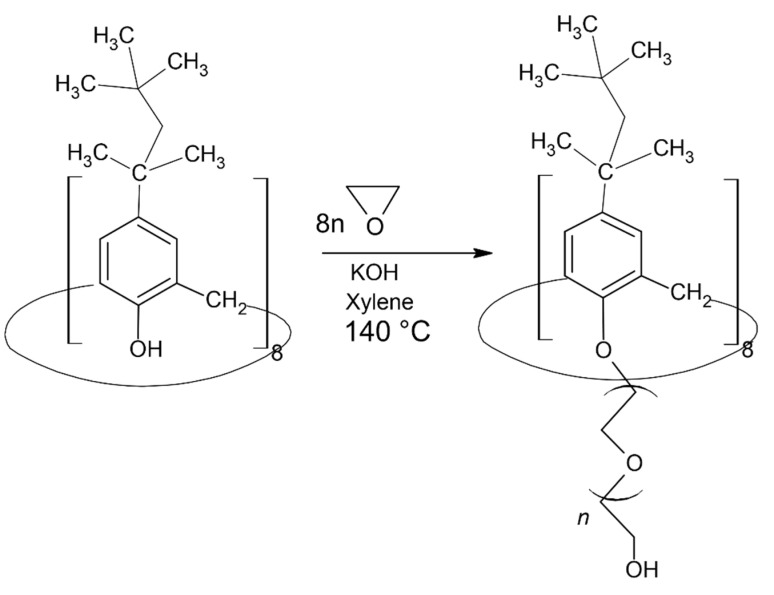
Schematic representation of the synthesis of PEGylated *tert*-octylcalix[8]arenes.

A series of PEGylated *tert*-octylcalix[8]arenes were synthesized via the “grafting from” approach. By varying the time of polymerization of ethylene oxide (EO), and hence the amount of EO, PEG chains of varying degrees of polymerization were grafted from the lower rim of the *tert*-octylcalix[8]arene macrocycle. The synthetic approach is schematically presented in Figure 2. It yielded polymers of molecular weight distribution (M_w_/M_n_), as assessed by gel permeation chromatography (GPC) in the 1.40–1.70 range. A small fraction of molecular weight of about 1100 was typically present in the GPC eluograms, which was eliminated after washing with water, to yield M_w_/M_n_ in the range 1.10–1.15 (see ESI, Figure S1). The resulting products were amphiphilic macromolecules, consisting of a hydrophobic *tert*-octylcalix[8]arene core and eight arms of hydrophilic PEG chains.

The polymerization degrees of the PEG fragments and the corresponding average molar masses of the obtained PEGylated *tert*-octylcalix[8]arenes were determined from the relative areas of the signals of the CH_2_ groups of the PEG fragments at 3.5–3.7 ppm, and the CH_3_ protons of the tert-octyl groups at 1.0 ppm. A representative ^1^H NMR spectrum is shown in the ESI (Figure S2). The abbreviations of the newly synthesized PEGylated *tert*-octylcalix[8]arenes, as well as theoretical and experimental degrees of polymerization (DP) of the PEG chains and the number average molar masses (M_n_) of the investigated products, are given in Table 1. Static light scattering (SLS) measurements of selected samples showed a very good correlation between the molar masses of the products determined by ^1^H NMR spectroscopy and SLS (see below and the ESI, Table S1, Figure S3.

The successful PEGylation of *tert-*octylcalix[8]arene was evidenced by measuring the DOSY spectra of the new materials in CDCl_3_. Figure S2 shows as an example the DOSY spectrum of sample OEC-IV, where the PEG units (around 3.7 ppm) and the *tert*-octylcalix[8]arene fragments (0.4–1.7 ppm, 6.9 ppm) show identical diffusion coefficients, indicating that they originate from the same molecules.

### 3.2. Aqueous Solution Properties

**Figure 3:**
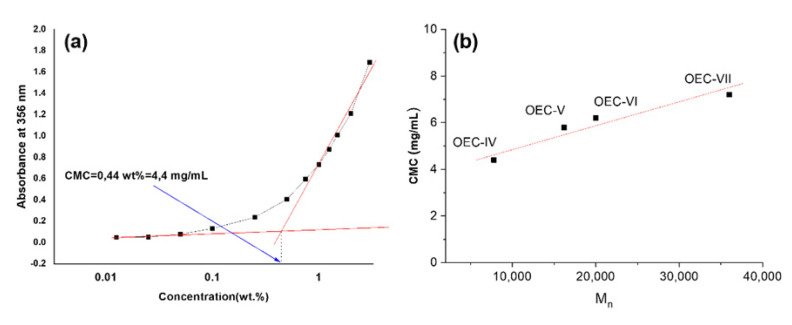
Absorption intensity at 356 nm and CMC determination of OEC-IV (**a**) and CMC versus molar mass of the investigated PEGylated *tert*-octylcalix[8]arenes in aqueous solution (**b**).

The lowest members of the series of PEGylated *tert*-octylcalix[8]arenes (OEC-I and OEC-II) were not soluble in water. OEC-III exhibited limited solubility at low concentrations, whereas the higher members (OEC-IV–OEC-VII) spontaneously dissolved in water in wide concentration intervals. Considering their non-linear chain topology, the possible steric hindrance caused by the densely functionalized PEG lower rim, and the screening of the hydrophobic moieties, one may anticipate more complicated and complex self-associating behavior compared to that of linear amphiphilic copolymers. The association behavior of the PEGylated *tert*-octylcalix[8]arenes in aqueous solution was investigated by a variety of methods, including dye solubilization, diffusion-ordered NMR spectroscopy, and light scattering. For determination of the CMCs, the sensitivity to changes in the microenvironment of the non-polar dye 1,6-diphenyl-1,3,5-hexatriene(DPH) was exploited [39,40,41,42,43]. Typically, an increase in the absorbance at 356 nm is associated with the formation of hydrophobic domains in which the dye is solubilized. Figure 3a shows a representative absorbance vs. concentration dependence for OEC-IV at 25 °C, from the break of which the CMC was determined. Similarly, the CMC values of all investigated species were determined. They fell in the range 4.4–7.2 mg/mL and showed a gradual increase with increasing M_n_ (Figure 3b). The lower CMC indicated easier and more favored self-association.

**Figure 4:**
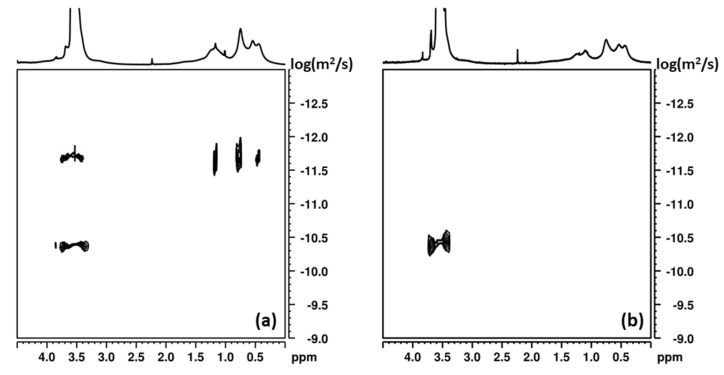
DOSY spectra of (**a**) OEC-III (10 mg/mL in D_2_O); (**b**) OEC-V (10 mg/mL in D_2_O).

Below the CMC, only unimers, that is, unassociated PEGylated *tert*-octylcalix[8]arenes, exist, whereas above the CMC multimolecular aggregates are formed. The transition from unimers to multimolecular aggregates, however, is not sharp, as evidenced by Figure 3a, which could be associated with the polymer nature of the products, their non-linear chain topology, and the presence of a rigid calixarene moiety, as well as some composition dispersity. In this relatively broad transition interval, unimers and multimolecular aggregates were found to co-exist, as evidenced by dynamic light scattering (see ESI, Figure S4 and DOSY. DOSY exploits the differences in the translational diffusion coefficients of various species present in a mixture, thus allowing discrimination between components with different sizes [44]. In the present study, it was used for determination of the diffusion coefficients and sizes of aggregates formed in aqueous solutions of the PEGylated *tert*-octylcalix[8]arenes, containing 14 (OEC-III), 17 (OEC-IV), 41 (OEC-V) and 96 (OEC-VII) oxyethylene units. The DOSY spectra of the systems with 14 and 96 oxyethylene units showed the presence of two components, indicating the formation of two types of aggregates. Figure 4a presents an example of the DOSY spectrum of OEC-III (10 mg in 1 mL D_2_O), showing the co-existence of relatively small particles with a diffusion coefficient of 3.38 × 10^−11^ m^2^/s and larger aggregates with a diffusion coefficient of 1.78 × 10^−12^ m^2^/s. The calculated apparent hydrodynamic diameter d_h_ of the former, of around 10 nm, could be associated with the size of unimers, while the latter, with a d_h_ of 190 nm, were undoubtedly multimolecular aggregates. Similar results were obtained for the system with 96 (OEC-VII) oxyethylene units (Figure S2 in the ESI). The lack of a diffusion peak for the calixarene fragment in unimers could be explained by the relaxation dynamics of the amphiphilic polymer system in water, which depends in a complex way on the overall and fragmental motion of the unimers and the aggregates. The systems with 17 and 41 oxyethylene units display only one component in their DOSY spectra corresponding to particles with relatively small sizes, and with a d_h_ of about 10 nm (Figure 4b). The good correlation between the sizes of the co-existing particles determined by DOSY and DLS (see Figure 4 and Figure S4) is noteworthy.

Similar behaviors, i.e., the co-existence of unimers and multimolecular aggregates in relatively wide concentration intervals, were observed for the other three water-soluble PEGylated *tert*-octylcalix[8]arenes.

Static light scattering (SLS) was employed to determine the molar mass and aggregation number of the multimolecular aggregates formed at concentrations well above the CMC. A representative Zimm plot is shown in Figure S3, and the derived parameters are collected in Table S1. The molar mass of the aggregates reached hundreds of kg/mol, corresponding to aggregation numbers (N_agg_) in the 11–20 range. These are considerably lower figures, corresponding also to the lower density of the materials within the particles, compared to the PEGylated calix[4]arenes studied earlier [45], and can be attributed to the larger size of the calix residue and the enhanced empty volume of the cavity resulting from the functionalization with the tert-octyl side groups at the upper rim.

### 3.3. Phase Solubility Evaluation

**Figure 5:**
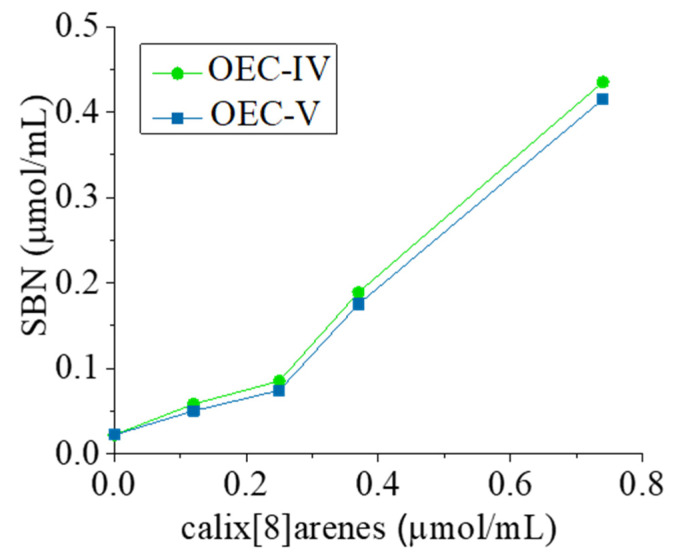
Phase solubility profiles of silibinin (SBN) in aqueous media containing various concentrations of OEC-IV (-●) or OEC-V (-■-) at 25 °C.

OEC-IV and OEC-V were selected for the preparation of platforms for the delivery of silibinin, and the further investigation and evaluation of their potential, because they are characterized by the lowest CMCs, and thus have enhanced stability upon dilution, larger N_agg_ (that is, larger hydrophobic volume and, hence, possibilities for loading greater drug amounts), and shorter PEG chains, which cause less spatial obstructions upon loading and the formation of inclusion complexes. The phase solubility of SBN in the presence of aqueous solutions of the investigated PEGylated tert-otctylcalix[8]arenes was determined by the method of Higuchi and Connors [46]. Due to their amphiphilic structure, OEC-IV and OEC-V can solubilize silibinin via two mechanisms: by the formation of inclusion complexes at concentrations below their CMC, and additionally by the formation of supramolecular aggregates at concentrations exceeding CMC. Therefore, the concentration range of the PEGylated calixarenes was selected to cover concentrations below and above their critical micellar concentration. The phase solubility profiles are shown in Figure 5.

As evident from the presented data, the gradual increase in the concentration of the PEGylated *tert*-octylcalix[8]arenes leads to an increase in the solubility of silibinin. Both solubility profiles can be defined as Ap type, as they show a positive deviation from linearity [47]. In the concentration range from 0.0 to 0.25 μmol/mL (concentrations below the CMC), a linear increase in SBN solubility is observed (R^2^ above 0.99). From the linear part of the solubility profiles, the slope of the lines can be derived (Table 2). Slopes less than 1 indicates the formation of “host–guest” inclusion complexes, between SBN and PEGylated *tert*-octylcalix[8]arenes, of a stoichiometric ratio of 1:1, following the equation:

(3) $$D+C\;\overset{Ks}{\Leftrightarrow }\left[DC\right],\;$$

where *D* is a guest drug molecule, *C* is the host macrocyclic compound and [*DC*] is the inclusion complex [46].

In addition, the slope values were used to calculate the stability constant (Ks), the main parameter describing the solubility of a drug and the stability of the complex, using the equation:

(4) $$\mathrm{Ks}=\frac{slope}{So\left(1-slope\right)}$$

where *So* is the solubility of silibinin in the absence of a complexing agent.

The Ks values are presented in Table 2. The calculated values for Ks are relatively high, which is an indicator of both the good solubility of silibinin and the sufficient stability of the complexes. In addition, inclusion complexes with Ks values above 100 are considered optimal for biological applications because, in addition to the optimized solubility of the drug, they also provide the ability for controlled release and, respectively, effective drug delivery in the target compartments [48,49]. For more in-depth characterization of the inclusion complexes, the Gibson free energy change of the complexation process was calculated following the equation:

(5) ΔG=−RTlnKs

Negative Δ*G* values (Table 2) indicate the spontaneous complex formation of SBN and the tested PEGylated *tert*-octylcalix[8]arenes in aqueous media.

Although there is no significant difference in the studied parameters between the OEC-IV and OEC-V inclusion complexes, there is a tendency towards the lower solubility of silibinin in the presence of OEC-V. A probable explanation is the spatial obstruction of the longer PEG chains, which may hinder the entry of SBN molecules.

At concentrations above the CMC, a positive deviation in the phase solubility profiles can be clearly seen (Figure 5), evidenced by the formation of supramolecular aggregates, in the hydrophobic domains of which additional amounts of silibinin can be solubilized, leading to a sharp increase in its aqueous solubility. The total solubility improvement of silibinin was studied at OEC-IV and OEC-V concentrations of 1.28 and 0.74 μmol/mL (above CMC), respectively, and was expressed as the solubility enhancement factor (δ), calculated by Equation (6) [47]:

(6) $$\delta =\frac{S-So}{So}\times 100$$

where *So* and *S* denote silibinin solubility in the absence and presence PEGylated *tert*-octylcalix[8]arenes, respectively.

The solubility enhancement factors are presented in Table 2 and are 100% when the solubility of silibinin exceeds twice its *So* (*S* = 2*So*). Thus, the addition of the tested PEGylated *tert*-octylcalix[8]arenes at concentrations far exceeding their CMCs leads to a more than 20-fold increase in the aqueous solubility of silibinin via two simultaneously occurring mechanisms: the formation of 1:1 inclusion complexes and the formation of supramolecular aggregates.

### 3.4. Characterization of OEC:SBN Inclusion Complexes

#### Fourier Transform Infrared (FT-IR) Spectroscopy

**Figure 6:**
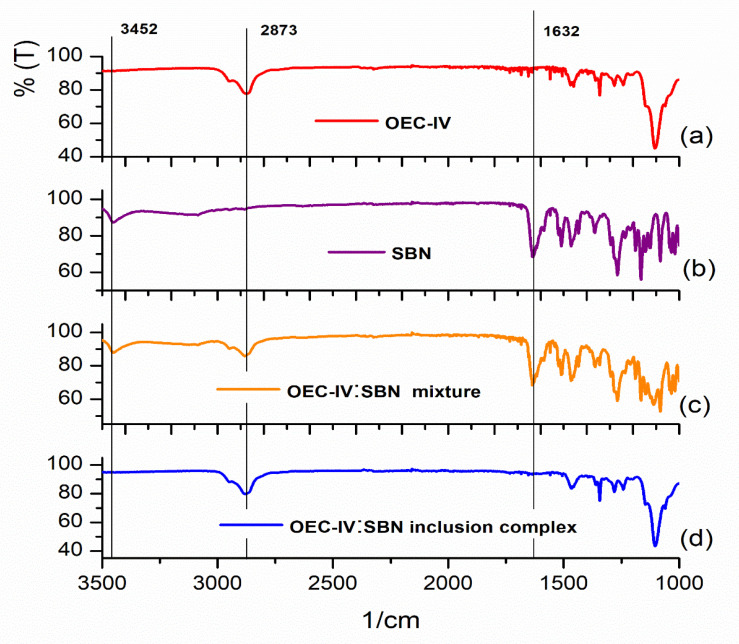
FT-IR spectra in the region 1000–3000 cm^−1^ of (**a**) OEC-IV, (**b**) silibinin, (**c**) the OEC-IV:silibinin physical mixture and (**d**) the OEC-IV:silibinin inclusion complex.

Representative FT-IR spectra of SBN, OEC-IV, the physical mixture of OEC-IV and SBN and the inclusion complex OEC-IV:SBN are shown in Figure 6. The results obtained for other OEC samples are quite similar. The spectrum of free SBN (Figure 6b) shows characteristic peaks at 3452 cm^−1^ (-OH stretching vibration), 1632 cm^−1^ (C=O stretching vibration) and 1506–1468 (skeleton vibration of aromatic C=C ring stretching) [50]. These did not interfere with the bands in the OEC-IV spectrum, and were used as marks for the description of silibinin in the inclusion complex. The characteristic peak at 2873 cm^−1^ in the spectrum of OEC-IV (Figure 6a) is caused by the asymmetric and symmetric stretching vibrations of the CH bonds in calixarene [51]. The FT-IR spectrum of the physical mixture (Figure 6c) is a combination of the spectra of pure SBN and OEC-IV, and shows the characteristic bands of both molecules. In contrast, the spectrum of the inclusion complex (Figure 6d) does not show SBN’s characteristic peaks, which is probably due to the restriction of the vibration of the SBN molecule. This observation suggests that the silibinin molecule was entrapped in the hydrophobic calixarene cavity.

### 3.5. Characterization of Silibinin-Loaded OEC Supramolecular Aggregates

#### 3.5.1. Size, Size Distribution and Zeta Potential

**Figure 7:**
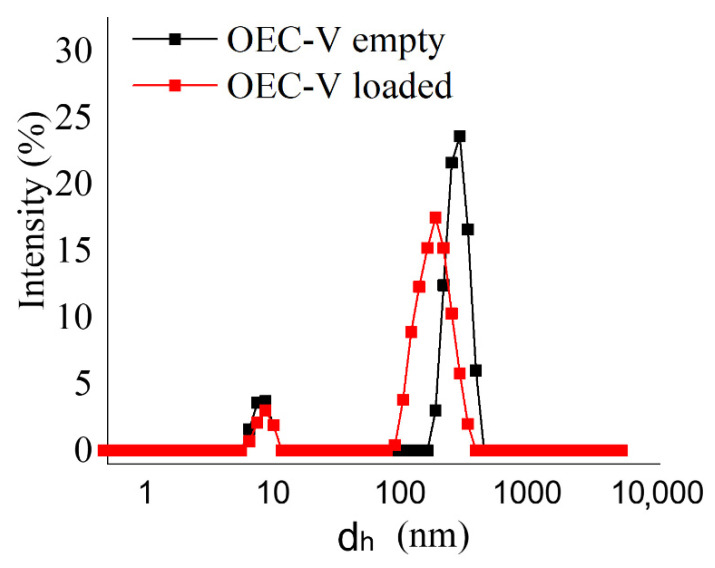
Size distribution curves of non-loaded and silibinin-loaded supramolecular OEC-V aggregates.

The size, size distribution patterns and ζ potential of the formed silibinin:OEC aggregates were measured by DLS and electrophoretic light scattering. The results are summarized in Table 3. Representative size distribution curves are depicted in Figure 7.

Evident in the presented results are the relatively high PDI values (Table 3) corresponding to the bimodal size distributions (Figure 7): a small fraction (less than 13%) of particles with a size around 10 nm (presumably unimers, see Section 2.2 above) was found to coexist with a dominant fraction (above 87%) of particles with sizes varying from 200 to 295 nm in all of the studied formulations, whether empty or silibinin-loaded. Given the very low percentage distribution of the concomitant fractions of particles and their small sizes, it can be concluded that the presence of these species would not affect the uniform loading of silibinin in the main group of supramolecular aggregates. Another interesting finding from the DLS analysis is that the silibinin loading in the aggregates is accompanied by a decrease in their size (Table 3). A possible explanation for the observed trend is the condensation of the hydrophobic core of the supramolecular aggregates as a result of the binding interactions between the molecules of silibinin and the hydrophobic domains of the calixarene aggregates. These observations are consistent with the findings of other authors who have also shown a reduction in the size of polymer micelles with the inclusion of hydrophobic drugs [52].

Although the PEGylated *tert*-octylcalix[8]arenes under investigation in the present study are nonionic amphiphiles, their supramolecular aggregates, both non-loaded and silibinin-loaded, show a relatively high negative ζ potential (Table 3), which is an indicator of their physical stability. On the other hand, the encapsulation of silibinin was associated with a substantial shift to less negative values. This shift in the ζ potential is probably due not only to the localization of silibinin molecules in the hydrophobic interior of supramolecular aggregates, but also due to their absorption on the surface as a result of the formation of hydrogen bonds between the ether oxygens of the PEG chains of PEGylated *tert*-octylcalix[8]arenes and the OH or keto groups of silibinin molecules. Nevertheless, the absolute value of the ζ potential of the loaded formulations remains relatively high, which is a prerequisite for sufficient physical stability.

#### 3.5.2. Silibinin Release Study

**Figure 8:**
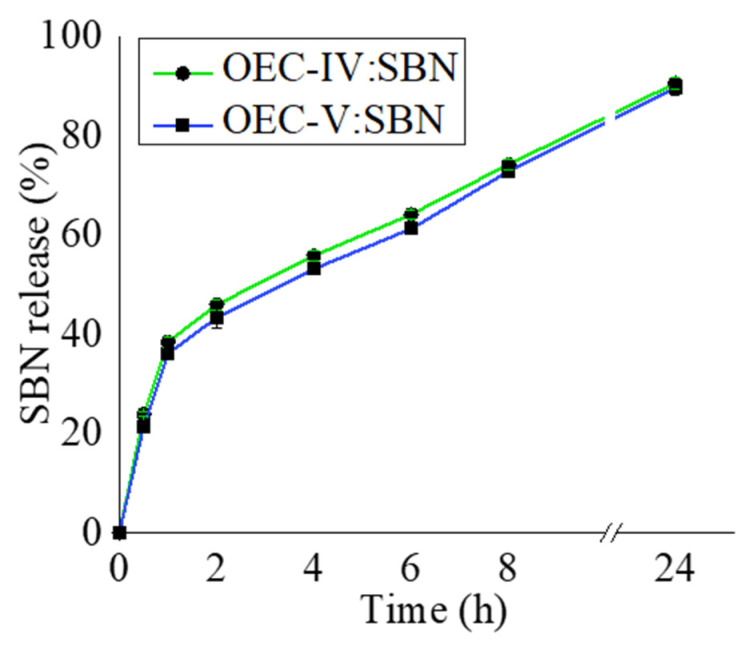
Release profiles of silibinin from supramolecular PEGylated *tert*-octylcalix[8]arenes aggregates.

The release behavior of silibinin from supramolecular OEC-IV and OEC-V aggregates was investigated via the dialysis technique against PBS (pH 7.4) at 37 °C for 24 h. The release profiles are shown in Figure 8. The presented results show the two-phase release profiles of silibinin from both types of aggregates. The initial “burst” release, where within 3 h almost 50% of the loaded substance was released, was followed by a delayed silibinin release up to the 24th h. These results are consistent with those of our previous studies of similar nanosized systems of curcumin delivery, which showed the same release behavior [23]. This finding confirms our hypothesis that the initial fast release was due to the release of silibinin from the aggregates, while the slower second phase was due to the release of silibinin from the inclusion complexes, which were characterized with relatively high values of Ks and, as such, mediated a slower drug release (see Section 3.3 and Table 2).

#### 3.5.3. Cytotoxicity Study

**Figure 9:**
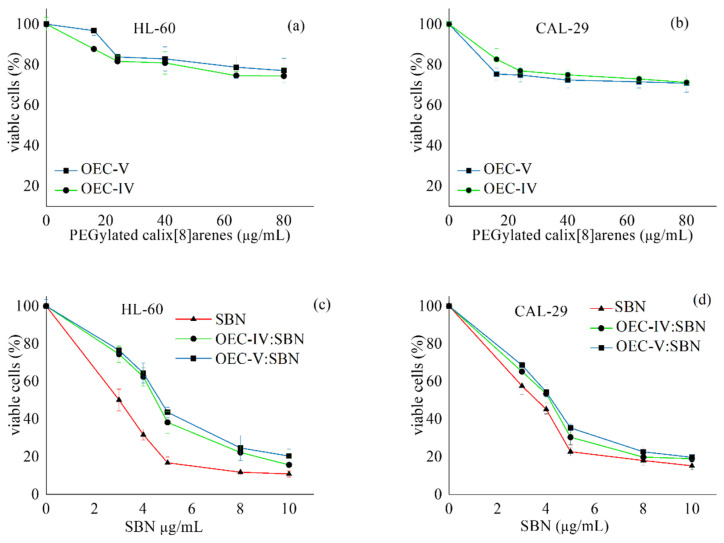
Cytotoxicity of non-loaded supramolecular PEGylated *tert*-octylcalix[8]arenes aggregates (**a**,**b**) and their silibinin-loaded counterparts (**c**,**d**) against human tumor cell lines HL-60 (**a**,**c**) and CAL-29 (**b**,**d**) after 72 h exposure at 37 °C, ±SD from 6 separate experiments.

The PEGylated *tert*-octylcalix[8]arenes formulations of silibinin were evaluated in comparison with the free drug for antineoplastic activity against chronic myeloid leukemia (HL-60)- and transitional cell urinary bladder cancer (CAL-29)-derived cell lines after of 72 h continuous exposure, using the MTT dye reduction assay. Both OEC-V and OEC-IV non-loaded systems were tested against these cell lines as well, and as evident from the concentration–response curves depicted in Figure 9a,b, they exerted only marginal intrinsic cytotoxicity. The comparative evaluation of the cell growth inhibition following treatment with the free drug or its formulations (Figure 9c,d, Table 4) showed strong, concentration-dependent cytotoxic effects, with IC_50_ values within the low μg/mL range. Although there was a shift in the dose–response curves towards higher concentrations in the formulated vs. free silibinin, the IC_50_ values thereof were comparable. This suggests that the PEGylated *tert*-octylcalix[8]arenes, although being generally devoid of intrinsic cytotoxicity, did not compromise the antineoplastic potential of the natural compound.

## 4. Conclusions

Novel PEGylated *tert*-octylcalix[8]arenes were designed as carriers of silibinin—an anticancer and chemo-protective agent with hepatoprotective properties and high therapeutic potential. The products were obtained by the “grafting from” approach. PEG chains with degrees of polymerization varying from 4 to 96 were grafted from the lower rim of the original *tert*-octylcalix[8]arene macrocycles to produce amphiphilic macromolecules consisting of a hydrophobic *tert*-octylcalix[8]arene core and eight arms of hydrophilic PEG chains. In an aqueous solution, the PEGylated *tert*-octylcalix[8]arenes were found to self-associate above a certain critical concentration into nanosized aggregates. The resulting supramolecular structures were used for the solubilization and delivery of silibinin. Tremendous enhancements in the solubility of silibinin (>1700%) were observed, and were attributed to the simultaneous formation of inclusion complexes and additional solubilization in hydrophobic domains of the supramolecular aggregates. Accordingly, two phases were observed in the release profiles of silibinin: fast release from the aggregates and considerably slower release from the inclusion complexes. The investigated PEGylated *tert*-octylcalix[8]arenes exerted only marginal intrinsic cytotoxicity, and did not compromise the antineoplastic potential of silibinin. Based on a recent review, with a detailed summary of various SBN formulations [53] and focusing on their favorable physico-chemical characteristics, ability to significantly enhance solubility, excellent biocompatibility, and appropriate release profiles, the PEGylated *tert*-octylcalix[8]arenes were found to further expand the experimental knowledge in this field, and can be considered as promising carriers for the delivery of silibinin.

## Figures and Tables

**Table 1:** Abbreviations, theoretical and experimental degrees of polymerization (DP) of the PEG chains and number average molar mass (M_n_) of the PEGylated *tert*-octylcalix[8]arenes.

Abbreviation	DP of PEG Chains		Mna
	Theoretical	Experimental ^a^	
OEC- I	5	4	3200
OEC-II	7	6	3900
OEC- III	19	14	6700
OEC-IV	22	17	7800
OEC- V	42	41	16,200
OEC- VI	57	52	20,000
OEC- VII	100	96	36,000

^a^ Derived from ^1^H NMR data in CDCl_3_.

**Table 2:** Stability constants (Ks), thermodynamic parameter (ΔG) and solubility enhancement factor (δ) derived from phase solubility diagrams. *So*—intrinsic solubility of SBN in the absence of complexing agents.

	Parameter	Slope	R^2^	Ks (mL/μmol)	ΔG (kJ/mol)	*So* (μmol/mL)	δ (%)
Complex							
SBN:OEC-IV		0.73556	0.998	126.4	−11.98	0.022	1877
SBN:OEC-V		0.73301	0.996	124.5	−11.84		1786

**Table 3:** Size, size distribution patterns and ζ potential of empty and silibinin-loaded supramolecular aggregates.

Sample	Diameter (nm)	PDI	ζ Potential (mV)
OEC-IV empty	260.0 ± 5.2	0.54	−32.2 ± 1.55
OEC-IV:SBN	211.0 ± 2.4	0.44	−23.1 ± 0.35
OEC-V empty	295.0 ± 3.8	0,48	−31.5 ± 0.5
OEC-V:SBN	200.0 ± 5.6	0.39	−19.9 ± 1.9

**Table 4:** IC_50_ values of free and loaded silibinin (μg/mL).

Sample	IC_50_	
	HL-60	CAL-29
SBN	3.01	3.61
OEC-IV:SBN	4.48	4.13
OEC-V:SBN	4.67	4.25

## Supplementary Materials

The following are available online: https://www.mdpi.com/article/10.3390/pharmaceutics13122025/s1. Figure S1: GPC eluograms of crude (a) and purified (b) OEC-VI. Figure S2: (a)Representative ^1^H NMR spectrum of OEC-VII in CDCl_3_ for determination of the average DP of the PEG chains and number average molar mass (M_n_).-; (b) DOSY spectrum of OEC-IV in CDCl_3_ (10 mg/mL); (c) DOSY spectrum of OEC-VII (10 mg/mL in D_2_O) showing the co-existence of two populations of particles—unimers and macromolecular aggregates. Figure S3: (a) Debye plot for OEC-VII in water at 25 °C in the concentration range below the CMC. Measurements were made at an angle of 90°; (b) Zimm plot for OEC-VII in water at 25 °C in the concentration range above the CMC. Figure S4: Particle size distribution from DLS of OEC-VII in water at 25 °C in the transition concentration range. Measurements were made at an angle of 90° and c = 6.2 mg/mL. Table S1: SLS characterization parameters of selected samples in water at 25 °C in the concentration ranges below or above the CMCs.
