# Supplementary material for: PEG-Modified tert-Octylcalix[8]arenes as Drug Delivery Nanocarriers of Silibinin
Source: Pharmaceutics. 2021 Nov 27;13(12):2025. doi: 10.3390/pharmaceutics13122025 (PMC8709077; doi:10.3390/pharmaceutics13122025)
Supplement: Supplementary file 1 [file pharmaceutics-13-02025-s001.zip › pharmaceutics-1448257-supplementary-second proof.pdf]

pharmaceutics

Supplementary Materials: PEG-Modified *tert*-Octyl-calix[8]arenes as Drug Delivery Nanocarriers of Silibinin

Desislava Budurova, Denitsa Momekova, Georgi Momekov, Pavletta Shestakova, Hristo Penchev and Stanislav Rangelov

Gel permeation chromatography (GPC). GPC analyses were performed on a Shimadzu Nexera HPLC chromatograph equipped with a degasser, a pump, an autosampler, an RI detector, and three columns: 10 µm PL gel mixed-B and 5 µmPL gel 500 Å and 50 Å. Tetrahydrofuran was used as the eluent at a flow rate of 1.0 mL/min and temperature of 40 °C. The sample concentration was 1 g/L and GPC was calibrated with polystyrene standards. Representative GPC eluograms of crude and purified OEC-VI are shown in Figure S1. Typically, for the higher members of the series (OEC-VI – OEC-VII) a bimodal molecular weight distribution (M_w/M_n) was observed corresponding to M_w/M_n in the 1.40–1.70 range (Figure S1a). The low molecular weight fraction, most probably homooligo(ethylene glycol), was removed by washing the crude product by water (Figure S1b). The typical M_w/M_n values of the purified PEGylated *tert*-octylcalix[8]arenes ranged in the interval 1.10–1.15.

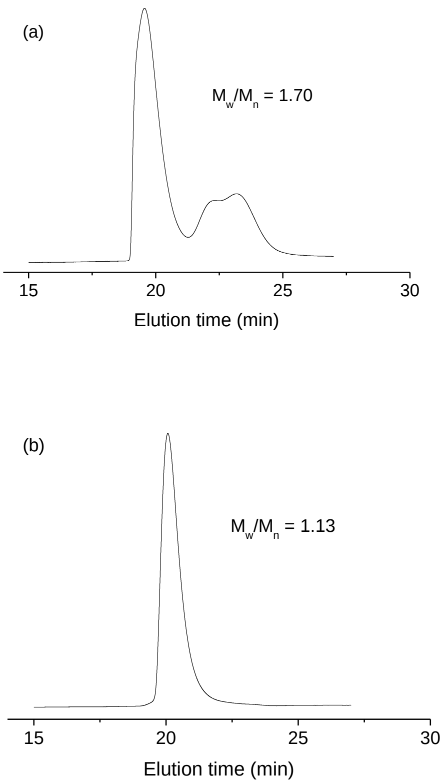

Figure S1. GPC eluograms of crude (**a**) and purified (**b**) OEC-VI.

^{1}H NMR and DOSY characterization. ^{1}H NMR spectra were recorded in $CDCl_3$ on a Bruker Avance II+ 600 MHz instrument. A representative ^{1}H NMR spectrum of OEC-VII in $CDCl_3$ is shown in Figure S2a. The degrees of polymerization of the grafted PEG chains and the corresponding number average molar masses of the resulting PEGylated *tert*-

octylcalix[8]arenes were determined from the ^{1}H NMR spectra using the relative intensities of the resonances of the methylene protons of the PEG moieties at 3.2–4.2 ppm and the methyl protons of the tert-octyl groups at 0.7 ppm.

Figures S2b and S2c show DOSY spectra of selected PEGylated *tert*-octylcalix[8]arenes in $CDCl_3$ and D_2O, respectively. The successful PEGylation and effective purification of the products were evidenced by the DOSY spectrum in $CDCl_3$ (Figure S2b). It is clearly seen that PEG and ***tert***-octylcalix[8]arene fragments have identical diffusion coefficients, indicating that they are constituent parts of one and the same macromolecule. The DOSY spectra of the systems with 14 and 96 oxyethylene units in D_2O (Figure S2c) show the presence of two components indicating the formation of two types of aggregates - unimers (unassociated macromolecules) and macromolecular aggregates.

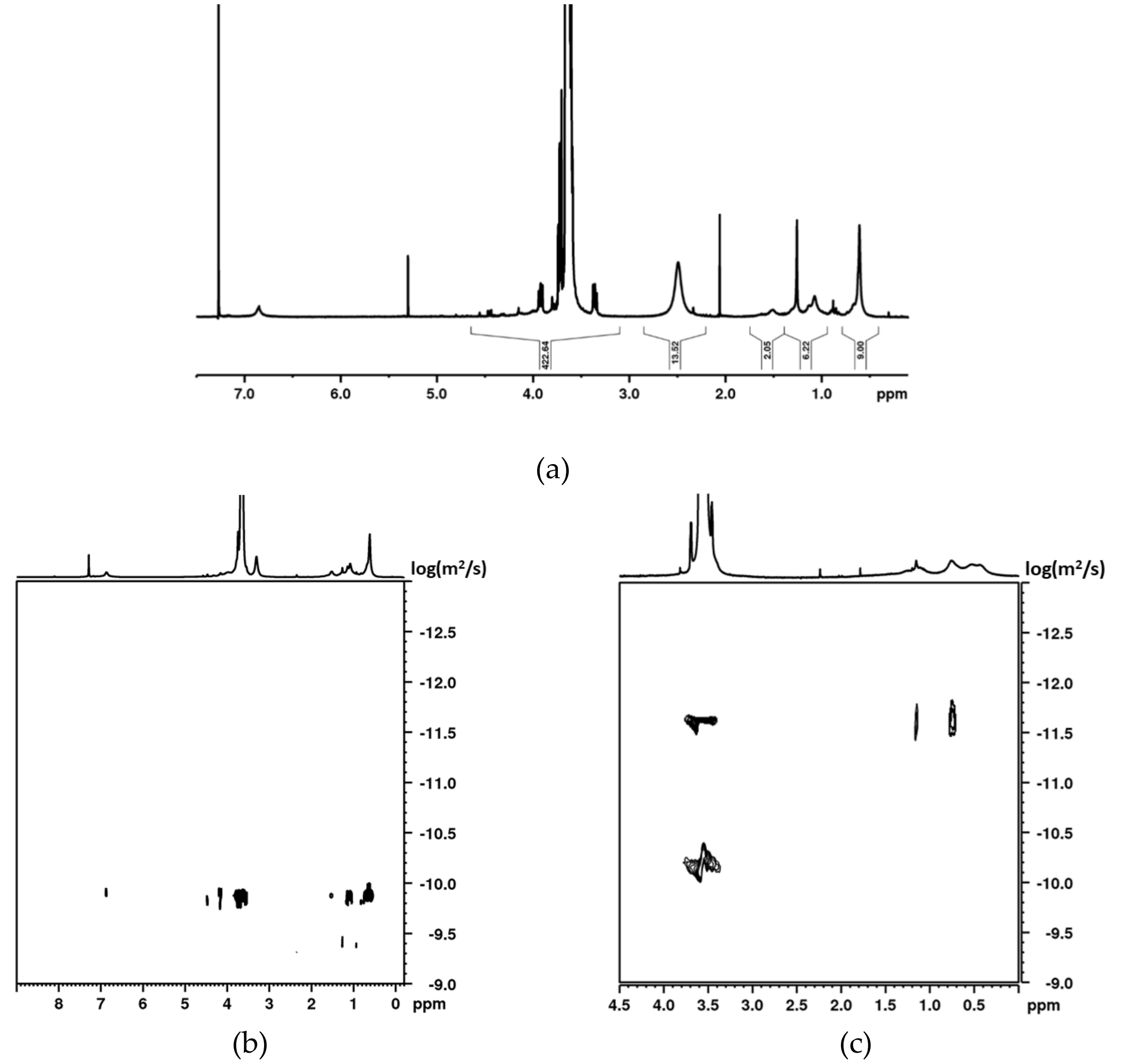

Figure S2. (**a**) Representative ^{1}H NMR spectrum of OEC-VII in $CDCl_3$ for determination of the average DP of the PEG chains and number average molar mass (M_n); (**b**) DOSY spectrum of OEC-IV in $CDCl_3$ (10 mg/mL); (**c**) DOSY spectrum of OEC-VII (10 mg/mL in D_2O) showing the co-existence of two populations of particles – unimers and macromolecular aggregates.

Static light scattering (SLS). The SLS measurements were carried out in the interval of angles from 40 to 140° at 25 °C using a Brookhaven BI-200 goniometer with vertically polarized incident light at a wavelength λ = 633 nm supplied by a He–Ne laser operating at 35 mW. The SLS data were analyzed using the Debye or Zimm plot softwares provided

by Brookhaven Instruments. Information on the weight-average molar mass, M_w, was obtained from the dependence of the quantity Kc/R_{90} on the concentration (c) at scattering angle θ=90° (Debye plot method) or from the dependence of the quantity $Kc/R_θ$ on the concentration (c) and scattering angle θ in the interval of angles from 40 to 140° (Zimm plot method). Here, K is the optical constant given by $K = 4\pi^2 n_0^2(dn/dc)^2/N_A\lambda^4$, where n_0 is the refractive index of the solvent (water), N_A is Avogadro's constant, λ is the laser wavelength, and $R_θ$ is the Rayleigh ratio at θ. dn/dc is the refractive index increment measured in water in separate experiments on an *Orange GPC19 DNDC* refractometer. The dn/dc values were in the 0.1352–0.1396 (mL/g).

The Debye plot method was applied for determination of the weight-average molar mass, M_w, of the unassociated (that is, in the concentration range below the CMC) PEGylated *tert*-octylcalix[8]arenes. Figure S3a shows a Debye plot for OEC-VII. The measurements were done at a single angle of 90°, because, due to the small size of the scattering particles (unassociated OEC-VII molecules, unimers), angular dependence of the quantity $Kc/R_θ$ was not observed. The derived value of weight-average molar mass (M_w = 38,000 g/mol, Figure S3a, Table S1) is in good correlation with M_n, determined from ^{1}H NMR (M_n = 36,000, see Table 1).

Weight-average molar masses of the multimolecular aggregates (that is, in the concentration ranges above the CMC) were evaluated by the Zimm plot method. A representative Zimm diagram is shown in Figure S3b. The values of M_w are collected in Table S1. The aggregation number, N_{agg}, was calculated from the light scattering data for molar mass of multimolecular aggregates (M_w^{agg}) and NMR data for number-average molecular weight of the unimers (M_n^{uni}) as Equation (1):

$$N_{aag} = M_w^{agg}/M_n^{uni} \quad (1)$$

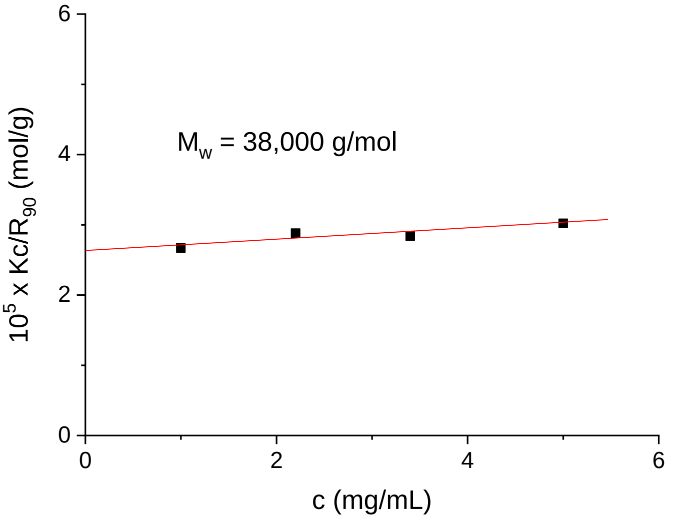

(a)

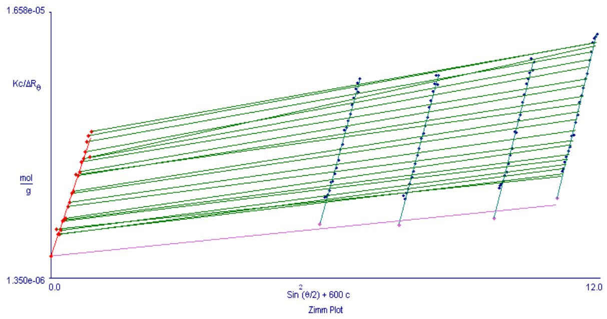

(b)

Figure S3. (**a**) Debye plot for OEC-VII in water at 25 °C in the concentration range below the CMC. Measurements were made at an angle of 90°; (**b**) Zimm plot for OEC-VII in water at 25 °C in the concentration range above the CMC.

Table S1. SLS characterization parameters of selected samples in water at 25 °C in the concentration ranges below or above the CMCs.

Sample Abbreviation	Concentration Range	M_w (g/mol)	N_{agg}	Comments	R_g (nm)	A_2 (ml·mol/g^2)
OEC-VII	Below the CMC	38,000	1	Unimers (unassociated molecules)	-	2.23×10^{-4}
OEC-IV	Above the CMC	1.52×10^5	20	Multimolecular aggregates	65.8	1.49×10^{-4}
OEC-VI	Above the CMC	3.2×10^5	16	Multimolecular aggregates	90.0	0.43×10^{-4}
OEC-VII	Above the CMC	3.8×10^5	11	Multimolecular aggregates	113.4	0.79×10^{-4}

Dynamic light scattering (DLS). DLS measurements were performed on a Brookhaven BI-200 goniometer with vertically polarized incident light at a wavelength λ = 633 nm supplied by a He–Ne laser operating at 35 mW and equipped with a Brookhaven BI-9000 AT digital autocorrelator. Measurements were made at an angle θ=90°. The autocorrelation functions were analyzed using the constrained regularized algorithm CONTIN [1] to obtain the distributions of the relaxation rates (Γ). The latter provided distributions of the apparent diffusion coefficient ($D = \Gamma/q^2$) where q is the magnitude of the scattering vector given by $q=(4\pi n/\lambda)\sin(\theta/2)$, n is the refractive index of the medium. The mean hydrodynamic diameter (D_h) was obtained by the Stokes–Einstein Equation (2):

$$D_h = kT/(3\pi\eta D) \quad (3)$$

where k is the Boltzmann constant, η is the solvent viscosity at temperature T in Kelvin and D is the diffusion coefficient. All measurements were performed at 25 °C at a single concentration.

Figure S4 shows a typical particle size distribution for the PEGylated *tert*-octylcalix[8]arenes in water at concentrations, which fall into the transition concentration range. The bimodal distribution is clearly observed. The two populations of particles that co-exist in this concentration range correspond to unimers (D_h of about 12 nm) and multimolecular aggregates (D_h of about 240 nm).

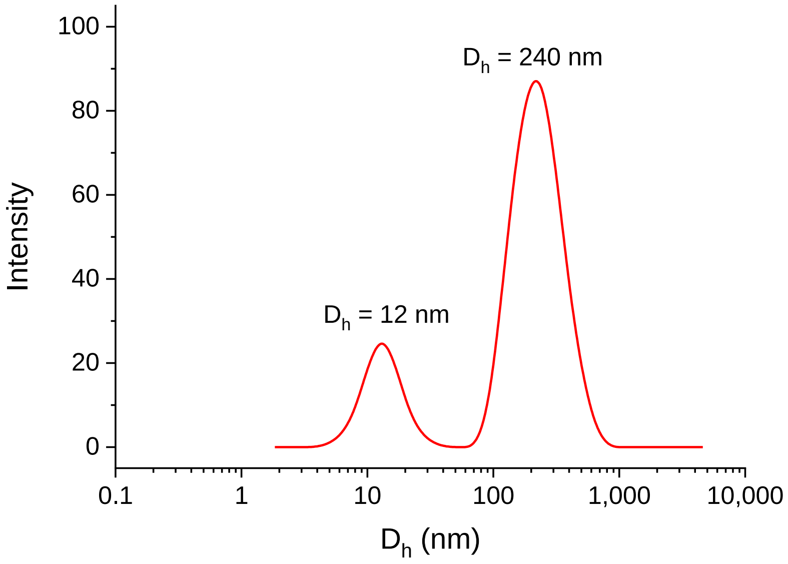

Figure S4. Particle size distribution from DLS of OEC-VII in water at 25 °C in the transition concentration range. Measurements were made at an angle of 90° and c = 6.2 mg/mL.

References

1 Provencher, S.W. Inverse problems in polymer characterization: Direct analysis of polydispersity with photon correlation spectroscopy. *Macromol. Chem.* **1979**, *180*, 201–209. DOI: 10.1002/macp.1979.021800119
